# Novel RNA extraction method from human tears

**DOI:** 10.22099/mbrc.2022.45266.1801

**Published:** 2022

**Authors:** Mahintaj Dara, Azam Habibi, Negar Azarpira, Mehdi Dianatpour, Mahmood Nejabat, Amir Khosravi, Nader Tanideh

**Affiliations:** 1 Stem Cells Technology Research Center, Shiraz University of Medical Sciences, Shiraz, Iran; 2 Department of tissue engineering and cell therapy, School of Advanced Technology in Medicine, Shiraz University of Medical Sciences, Shiraz, Iran; 3 Transplant Research Center, Shiraz University of Medical Sciences, Shiraz, Iran; 4 Department of Medical Genetics, Shiraz University of Medical Sciences, Shiraz, Iran; 5 Poostchi Ophthalmology Research Center, Department of Ophthalmology, School of Medicine, Shiraz University of Medical Sciences, Shiraz, Iran

**Keywords:** RNA, Extraction method, Human tears, Biomarkers

## Abstract

Human tears can be used as a noninvasive source of genetic materials and biomarkers in the prognosis and diagnosis of ocular and non-ocular diseases. The present protocol is a novel direct RNA extraction method from tears. This study aims to provide a suitable method for direct extraction of RNA from tears with high quality and quantity. In this study, we develop a TRIzol base protocol for direct RNA extraction from human tears. quality and quantity of extracted RNA measured by calculation of 260/280 UV absorption ratio using Nanodrop and real-time PCR. RNA was extracted with this modified method and a purified (260/280 UV absorption ratio between 1.8 to 2 and a high yield of total RNA, on average 95 μg, from tears was extracted. In conclusion, we developed an easy and suitable method for direct extraction of total RNA from tears with high quality and quantity.

## INTRODUCTION

The use of body fluids and the analysis of their gene expression profile can be used as a non-invasive method in the prognosis and diagnosis of ocular and non-ocular diseases [1-4]. Tears are clear fluids that contain electrolytes, proteins, lipids, and small molecular metabolites that are in constant exchange with ocular surface epithelial cells and reflect corneal biochemistry and physiology. This fluid covers the surface of the eye and is responsible for the exchange of oxygen, nutrients and waste materials, protecting the eye from pathogens and healing damage. Therefore, tear analysis can be very important for monitoring eye health and disease [5]. 

Among body fluids such as blood and urine, tear fluid is possibly one of the most understated in terms of clinical value. As we move into the era of personalized, preventive medicine and health technology, tear analysis has unique advantages to become the next routine test of body fluids – in the clinic and beyond. To this end, recent breakthroughs as well as critical analytical and biochemical challenges are discussed [5]. The human tears is a source of genetic materials and biomarkers [6, 7]. Examining changes in the expression of genes and microRNAs in the tears of healthy and sick people is one of the ways to discover biomarkers related to diseases. To analyze and compare gene expression changes in patients and healthy people, RNA or microRNA should be extracted from tears [8, 9]. Considering the small amount of RNA in tears, it is very important to use a suitable extraction method that can extract RNA with high quality and quantity. This study aims to provide a suitable method for direct extraction of RNA from tears with high quality and quantity.

## MATERIALS AND METHODS

All of the study designs and test procedures were performed in accordance with the Helsinki Declaration II. The protocols were approved by the ethics committee of Shiraz University of Medical Science, Shiraz, Iran**.**

**Materials and Reagents: **Solution TRIzol™ Reagent (Thermo Fisher Scientific)**, **Chloroform for analysis EMSURE® ACS, ISO, Reag. Ph Eur (Merck)**, **Ice cold 70 % ethanol (Sigma-Aldrich), DEPC-treated Water (Thermo Fisher Scientific)**, **Isopropanol (Sigma-Aldrich) 

**Equipment: **Sampler micropipette set**, **RNase-free tips**, **RNase-free microfuge tubes (0.2 mL)**, **RNase-free micro tubes (1.5 mL)**, **Sterile plastic capillary tubes,Water bath, Refrigerated centrifuge

**Procedure: **Twenty volunteers participated in this project. Tear samples were collected from each person with an approximate volume of 20 microliters. The tears were collected with 10 μl plastic capillary tubes from the lower conjunctival fornix without anesthesia or stimulation. Then tears samples were placed gently into a 1.5-ml RNase-free microtubes and froze at -70 C for storage. 700 μl ice-cold TRIzol™ solution (Thermo Fisher Scientific) was added to the tube samples, vortexed for 5-10 sec, and incubated at room temperature for three min. 200 μl of chloroform ( EMSURE® ACS, ISO, Reag. Ph Eur. Merck) was added to samples, and mixed well for 10 sec by shaking. The samples were incubated on ice or 4^o^C for 5 min and centrifuged at 12000 rpm at 4^o^C for 15 min. Then the aqueous phase was transferred to a new RNase-free 1.5 ml tube (The three-formed phases are very loose. Be careful not to mix the aqueous phase with the middle phase that contains DNA). An equal volume of the liquid phase plus 100 microliters of isopropanol (Sigma-Aldrich) was added to the sample tubes, gently mixed and incubated at -20^o^C for 45 min. Then the mixture was centrifuged at 12000 rpm at 4 ^o^C for 15 min. The supernatant was discarded gently and added 1 ml of ice-cold 70% ethanol (Sigma-Aldrich). After that centrifuged at 4^o^C at 7500 rpm for 8 min. The supernatant was discarded gently and let the pellet at room temperature for a few minutes to dry. The pellet was dissolved in 30 to 50 μl of DEPC-treated water (Thermo Fisher Scientific). To better dissolve the pellet, the tube was placed in a 55-60°C water bath for 5 min.

**Real-time PCR: **To check the quality and quantity of the extracted RNA, real-time PCR was performed. The cDNA was synthesized with Add Script cDNA Synthesis Kit (Add-bio, Korea). For RT–PCR 0.5 μg of total RNA and oligo (dT) primer was used following the suppliers. RT reactions were performed at 25°C for 15 min; 50 min at 65°C and 80°C for 5 min. cDNA (1 μl) was used as a template for real-time PCR. We designed a set of primers for gapdh and beta-actin genes that were carried out as an internal standard control to assay the extracted RNA quantity. Two technical and biological replicates of each PCR reaction were used.

PCR reactions were performed in a total volume of 10 μl; 5 μl RealQ Plus 2x master mix green (Ampliqon), 3 μl distilled water, 0.5 μl forward primer, 0.5 μl reverse primer, and 1 μl cDNA. Then real-time PCR was optimized to determine the number of cycles that would allow product detection within the linear phase of mRNA transcripts amplification. The conditions for the reaction were as follows: Holding stage: initial 12 min denaturation step at 95°C, cycling stage: 15 s at 95°C, 1 min annealing, and extension step at 60°C, for 40 cycles.

## RESULTS AND DISCUSSION

**Figure 1:**
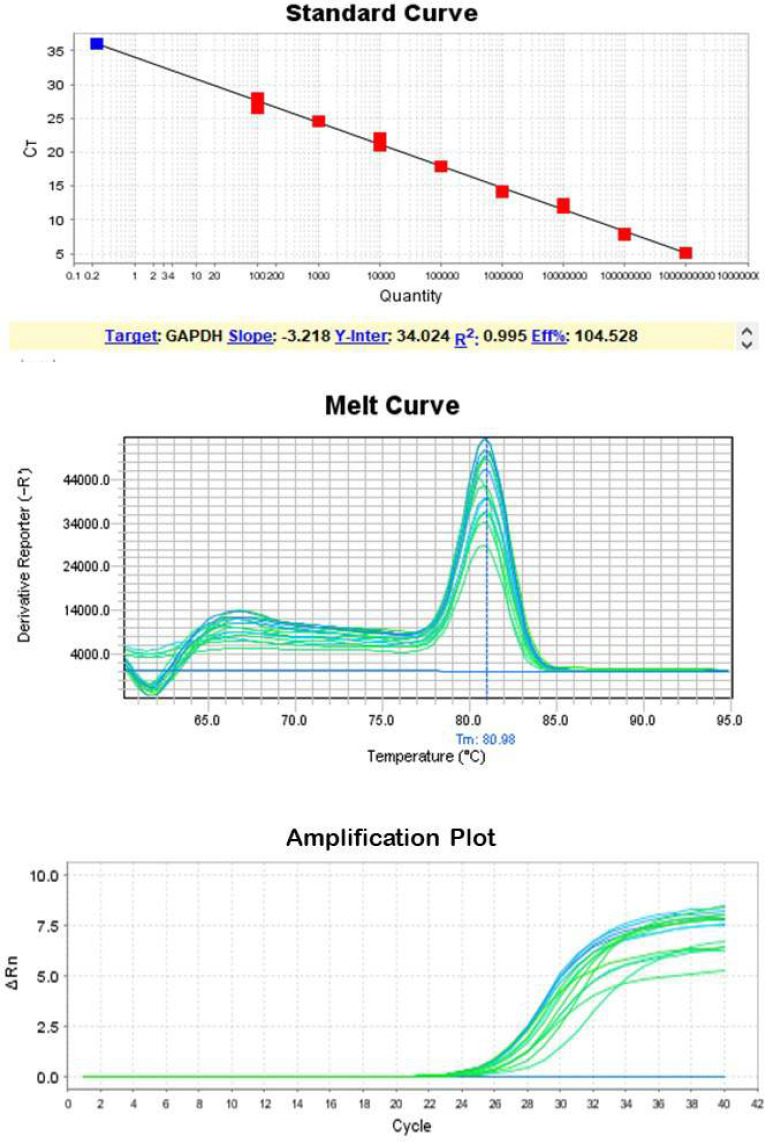
Standard Curve, Melt Curve and Amplification plot for Gapdh

**Figure 2:**
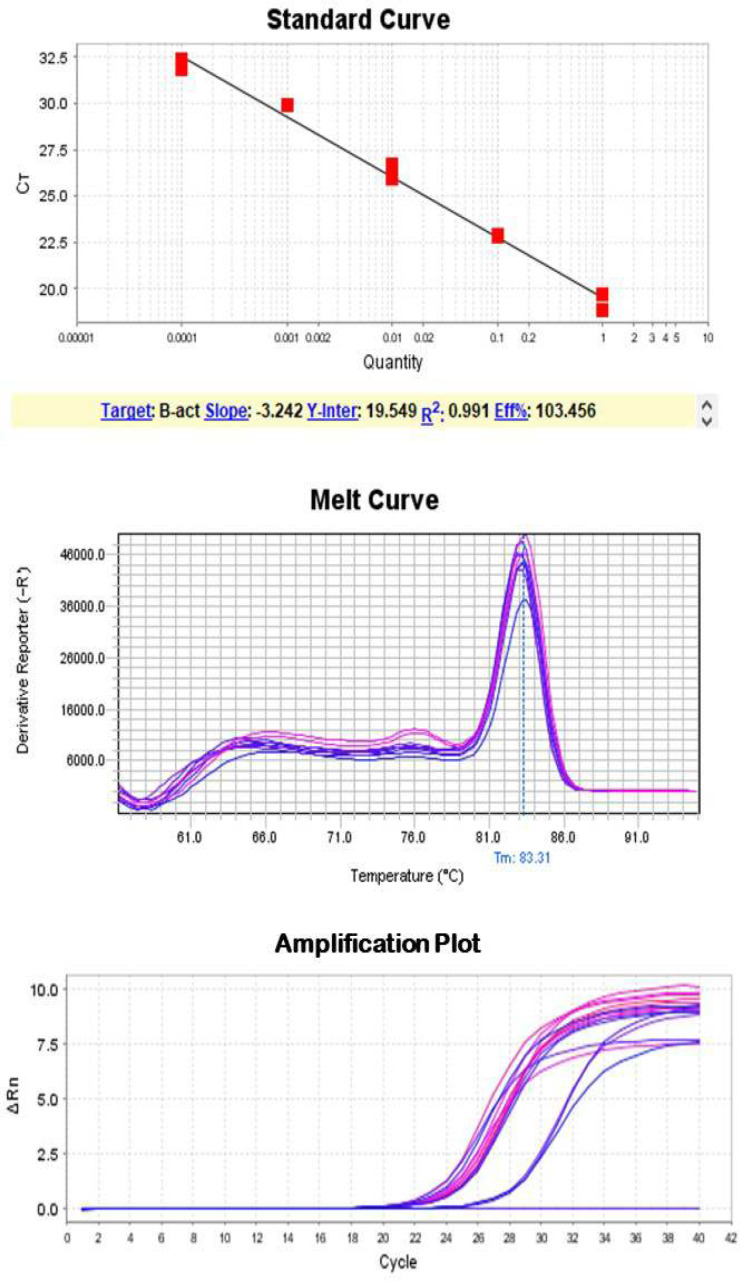
Standard Curve, Melt Curve and Amplification plot for Beta-actin

RNA was extracted with this modified method and a purified (260/280 UV absorption ratio between 1.8 to 2 and a high yield of total RNA, on average 95 μg, from tears was extracted (Table 1). Real-time PCR analysis showed that most samples have Ct values between 24 and 30 (Table 1), which is RNA of sufficient quality to provide reproducible results in gene expression assays (Fig. 1 & 2).

**Table 1:** RNA extracted concentration from each sample

**Sample ID**	**RNA Concentration in ** **40 μL DEPC Water** **(Nano gram/microliter)**	**UV absorption index (260/280)**	**Ct values for GAPDH**	**Ct values for Beta-Actin**
1	90	1.99	24.23	25.01
2	118	1.90	24.45	24.33
3	94	2.01	24.89	24.03
4	89	1.89	25.0	26.11
5	91	2.00	27.2	25.98
6	75	1.98	30.07	28.92
7	105	1.98	24.67	25.90
8	70	1.97	26.9	27.13
9	105	2.00	25.11	26.52
10	88	1.96	29.77	28.45
11	101	1.98	29.43	29.1
12	107	1.97	27.32	26.57
13	96	2.0	26.81	27.11
14	90	1.89	29.67	27.61
15	111	1.99	24.71	25.07
16	90	1.97	26.03	27.26
17	102	1.94	27.72	26.33
18	95	1.97	25.06	24.8
19	81	1.98	30.1	28.78
20	106	2.02	27.89	25.90

Biomarkers are molecules within the body that are capable of providing information according to the current physiologic state of a living organism like normal biological processes, pathogenic processes, or pharmacologic responses to therapeutic intervention [2, 10, 11]. Recently tears have been more considered as a potential source of biomarkers for prognostic or diagnostic purposes [12, 13]. Previously, tears exosomes have been used for the diagnosis of eye diseases [14-16]. Some studies used commercial kits for extraction of Total RNA or microRNA from tears exosomes [17] but in this study, we developed an easy TRIzol base protocol for direct RNA extraction from human tears with high quality and quantity. Easy and direct extraction of RNA in sufficient quantity and suitable quality can pave the way for further studies on tears as a non-invasive source of biomarkers.

## Acknowledgements:

The authors would like to thank Ms. Firoozeh. Dara for improving the use of English in the manuscript.

## Conflict of Interest:

Authors have no competing interest to disclose.
